# Cardioprotective effects of CoQ10 in pediatric patients with lysosomal storage disorders

**DOI:** 10.1186/s13052-025-02008-5

**Published:** 2025-05-28

**Authors:** Yasmine Abuzaid, Ghada Abdul Moemn Suliman, Osama Abd Rab El Rasool Toulba, Ahmed Abd El Basset Abo Elezz, Alshimaa Badraldeen, Heba Dawoud

**Affiliations:** 1 Pediatrics Department, Faculty of Medicine, Tanta University, Tanta, Egypt; 2 Clinical Pathology Department, Faculty of Medicine, Tanta University, Tanta, Egypt

**Keywords:** LSDs, NT-proBNP, MDA, ROS, STE, CoQ10

## Abstract

**Background:**

Lysosomal storage disorders (LSDs) result from an accumulation of specific substrates, due to the inability to break them down leading to cellular dysfunction in multiple organs, including the heart constituting an important and treatable cause of cardiomyopathy. Given the role of oxidative stress in many inborn errors of metabolism, many studies are evaluating oxidative stress and hence the role of antioxidants in patients with LSDs. The aim of this study: was to study the possible effects of Coenzyme Q10 as a cardioprotective and an antioxidant drug in patients with LSDs.

**Methods:**

This study was a prospective case-control study conducted on 30 patients with LSDs and an equal number of healthy subjects of matched age and sex served as a control group at the unit of Medical Genetics and inborn errors of metabolism at the Pediatric Department of Tanta University Hospital. All subjects included were subjected to full history taking, complete physical examination, assessing serum level of N-terminal pro-brain natriuretic peptide (NT-proBNP) & serum malondialdehyde (MDA), along with comprehensive cardiac evaluation that was done using tissue doppler imaging and speckling tracking echo. Then the patient group was subdivided into two subgroups, half of the patients received Co enzyme Q10 (CoQ10) while the other half received a placebo for 24 weeks followed by cardiac evaluation and reassessment of serum MDA and NT-proBNP for both patient subgroups.

**Results:**

Patients with LSDs had significantly higher levels of serum MDA than controls denoting higher oxidative stress, *P*-value < 0.001. CoQ10 resulted in significant beneficial reduction of serum MDA (15%) &NT-proBNP (30%) in the group of patients who received CoQ10, *P*-value < 0.001 and improvement of cardiac functional parameters in patients with LSDs.

**Conclusion:**

These findings suggest that CoQ10 may have a role in reducing oxidative stress and so may prevent the development of cardiomyopathy in patients with LSDs.

**Trial registration:**

This study was performed after approval from the Ethical Committee, Faculty of Medicine, Tanta University, Egypt (approval code 33255/07/19) and after obtaining written informed consent from children guardians. Also, this trial was registered on Pan African clinical trials registry with the number PACTR, ‘PACTR202107466690046’. Registered 04 July 2021.

## Background

LSDs are due to inherited mutations in genes that mainly encode lysosomal proteins. These mutations lead to defects in lysosomal targeted enzymes that degrade their specific substrates or defects in the proteins that regulate the delivery of these enzymes to the lysosome. The resulting phenotype usually associated with LSDs results from the abnormal accumulation of undegraded substrates leading to cellular dysfunction in multiple organs including the brain, heart, muscle, bone, skin, and spleen [1] [2, 3].

LSDs are classified according to the substrates they accumulate. These consist of mucopolysaccharidosis, mucolipidosis, sphingolipidosis, oligosaccharidosis, lipoprotein storage disorders, and neuronal ceroid lipofuscinoses along with others, up to 50 have been reported [4]. 

LSDs affect the heart and constitute a distinct and treatable cause of cardiomyopathy in pediatrics. Cardiac manifestations are a common finding in many LSDs and may range from obvious structural malformations (cardiomyopathy, cardiomegaly, cardiac valve anomalies, coronary artery disease) to more subtle functional issues including arrhythmias and conduction anomalies. LSDs affect the heart and constitute an important distinct and treatable cause of cardiomyopathy in children, accounting for about 5% of pediatric cardiomyopathies. Cardiovascular involvement is a significant cause of mortality and morbidity in patients with LSDs [5–8]. 

Metabolites accumulated in LSDs trigger a variety of pathogenic intracellular cascades, such as inflammation, oxidative stress, altered lipid trafficking, and autophagy ending in cell and tissue dysfunction. This induces excessive production of reactive species and/or depletes the tissue’s antioxidant capacity leading to damage to biomolecules (lipids, proteins, and DNA).

Considering the harmful effects caused by the excess of oxidative species in the cells, the use of antioxidants like CoQ10 as adjuvant therapy for some LSDs has been attracting increasing interest recently as it may provide some protection to this organelle from free radical-induced oxidation [9, 10]. 

Coenzyme Q10 (CoQ10) is a potent lipophilic antioxidant, exerting its antioxidant effect either by direct reaction with free radicals or indirect by regeneration of the antioxidants like vitamin C and vitamin E respectively from their oxidized state [11, 12]. 

CoQ10 is currently considered a potential experimental drug for the treatment of neurodegenerative diseases in general and lysosomal diseases in particular. It was shown that early long-term treatment with CoQ10 is cardioprotective and improves exercise performance in some systemic diseases in the pediatric age group [13–16]. 

## Patients and methods

### Study population and design

This randomized controlled trial was conducted at the units of Inborn Errors of Metabolism and Cardiology starting at the Pediatric Department of Tanta University Hospital (TUH) for a period of 2 years started from August 2019 to August 2021. Denoting the rare nature of LSDs, this study was conducted only on thirty patients diagnosed and followed with an LSD at the IEM Unit at the Pediatric Department of TUH during the period of the study and an equal number of healthy children of matched age and sex without any known chronic disorder served as a control group.

#### Sample size is 30 patients (it was calculated using G*Power 3.1.9) with effect size 0.15, alpha error 0.05 and power 85%

Enzyme assay for LSDs included in the study was done and all were below cut-off value for each disease and the patient with free sialic acid storage disease had high level of free sialic acid in urine along with clinical symptoms. Molecular study was done for some patients.

All subjects included were subjected to full history taking such as age, sex, the presence of similar conditions and positive consanguinity, complete physical examination and laboratory investigations in the form of serum N-terminal proBNP and MDA. Serum N-terminal proBNP was performed by ELISA kits (Shanghai Sun Red biological technology company, China) [17]. Plasma MDA was estimated by ELISA using Human MDA ELISA Kit supplied by Sun Red Shanghai Biological Technology Company [18].

#### Test principle

The kit uses a double-antibody sandwich enzyme-linked immunosorbent assay (ELISA) to assay the level of Human MDA and the level of Human (NT-proBNP) in samples.

The patient group was divided into two subgroups. Subgroup(a): 50% of patients received Coenzyme Q10 at a dose of (2 mg/kg daily) similar to a study performed by Pfeffer G. et al., and subgroup (b): The other half of patients received a placebo for 24 weeks, we choose this period (24 weeks) similar to another clinical trial study for the evaluation of the cardioprotective effects of captopril, simvastatin, and L-carnitine on the cardiac function in the diabetic children [19, 20]. 

Random allocation software was used for the generation of random sequences. Allocation concealment was performed by sequentially numbered sealed opaque envelopes. After signing the written consent, the sealed opaque envelope was opened, and the child was enrolled into the respective group. The randomization was performed by random number generation. All treating staff and outcome assessors were blinded to the treatment groups.

Serum NT-proBNP and MDA and echocardiographic measurements were performed 24 weeks after the treatment for all included patients to evaluate the effects of the given drug on cardiac parameters and laboratory investigations. We followed the participants by weekly phone calls and/or face-to-face meetings during enzyme replacement therapy sessions to provide the study materials and to take the used packages of drugs to ensure compliance with treatment. In addition, the parents were instructed to record any side effects during the treatment period.

This study was carried out on 30 patients with LSDs, but unfortunately, 4 patients dropped off during the study; one patient died, and three patients didn’t continue the follow-up due to personal issues. Two of the dropped patients were from the group that received CoQ10 while the other two patients were from the group that received a placebo hence each group consisted of 13 patients.

### Inclusion criteria

Pediatric patients with a lysosomal storage disease aged between 1 and 18 years and were diagnosed by enzyme assay or molecular study for LSDs.

### Exclusion criteria

Patients with congenital heart diseases, symptomatic cardiac complications, and associated systemic diseases like diabetes mellitus, cancer, and bronchial asthma were excluded as these diseases may cause cardiac diseases, alter the patient’s response to the used drug, and may affect the level of the studied biomarkers.

#### Equipment

Machine: Echocardiographic studies was performed using a commercially available ultrasound transducer and equipment (Vivid 7 and 9, GE Healthcare, Horten, Norway).

Transducers: Data acquisition was performed with a 3.5-MHz transducer, S7, and V3 matrix real-time 3-dimensional probes.

Workstation: Digital loops was stored on the hard disk of the echocardiography machine, and transferred to a workstation (Echo PAC PC, 112 and 113; GE, and Horten, Norway) for offline analysis.

### Measures

Full echocardiography examination with an experienced pediatric cardiologist was carried out for all included children using (Vivid 7 and 9; GE Healthcare, Horten, Norway) with 3.5 MHz, S7 and V3 matrix real-time three-dimensional probes, to assess cardiac function. Digital loops were stored on the hard disc of the echocardiography machine and transferred to a workstation (Echo PAC PC, 113; GE Healthcare) for offline analysis. Conventional echocardiography and Doppler examinations were performed to measure left ventricular fraction shortening (LV FS), LV ejection fraction (LV EF), LV end diastolic diameter (LVEDD), LV end-systolic diameter (LVESD), interventricular septal thickness, peak early diastolic filling velocity (E wave), peak late diastolic velocity (A wave), and early to late diastolic trans-mitral flow ratio (E/A). Where LV FS=(LVEDD − LVESD/LVEDD)×100 and LV EF= (LVEDD3 − LVESD3 /LVEDD3 )×100, Tissue Doppler imaging (TDI) was used to assess the systolic myocardial velocities at the basal segments of the lateral and septal walls (S) as well as the LV diastolic function by measuring the early and late diastolic myocardial velocities and their ratios (E′, A′, and E′/A′, respectively). The myocardial performance index (MPI) of LV was also measured by dividing the isovolumic time by the ejection time. We adjusted the frame rate between 100 and 150 frames/s for obtaining the optimal myocardial tissue images.

S wave at MV represented LV systolic function, while E′/A′ ratio at MV represented LV diastolic function. The myocardial performance index (MPI) could assess both systolic and diastolic ventricular function; it is the sum of the isovolumic contraction and the isovolumic relaxation time divided by the ejection time. Twodimensional speckle tracking echocardiography First, select images of the apical 4-chambers, the apical 2-chambers, and the apical 3-chambers views, and then we enter the 2D-strain mode under the Q-analysis, respectively, and manually trace the left ventricular endocardium border at the end of systole. After that we adjusted the endocardial envelope winding accurately and ensure the region of interest (ROI) contains complete ventricular wall information. Epicardial tracking was performed by the computer automatically. Tracking can be adjusted manually to increase tracking quality if needed. Finally, click the approve key to confirm the tracking. The software calculates the left ventricular global longitudinal strain (LV GLS) automatically [21]. We adjusted frame rate between 60 and 90 to capture the optimal myocardial tissue definition. For all measures, the mean of three consecutive cardiac cycles was taken.

3D global strain derives several parameters including longitudinal, circumferential, area and radial strain. Strain is the percent change in myocardial length or thickness from its original value. Positive values were obtained if regional thickening or lengthening occurred, and negative values were obtained if thinning or shortening occurred. Global area strain (GAS) was defined as the percentage change in the surface area which is defined by the circumferential and longitudinal strains vectors. The mean time taken to obtain strains by both 2D-STE and 3D-STE were also measured.

The three-dimensional full volume of the left ventricle was obtained during breath hold to assess left ventricular ejection fraction. The three-dimensional strain tracking is performed starting from a region of interest defined at end systole. The three-dimensional strain of the region of interest is automatically generated in the end-systolic frame and is built up from an endocardial and an epicardial mesh. The user can correct the region of interest manually for a better image [22, 23]. 

### Endpoints

The aim was to evaluate the elevated oxidative stress in patients with LSDs and evaluate the role of CoQ10 in reducing this oxidative stress and its cardioprotective effect.

### Statistical analysis

Data was analyzed using IBM SPSS software package version 20.0. (Armonk, NY: IBM Corp). Qualitative variables were presented as numbers and percentages and were compared by Chi-square test. Quantitative variables were presented as means ± standard deviation if normally distributed. Normal distribution of the data was checked by Shapiro–Wilk test. One-way analysis of variance was used to compare normally distributed quantitative variables between groups. Paired t-test was used to compare quantitative variables within the same group before and after the treatment. Quantitative variables without normal distribution were expressed as median and interquartile range (IQR) and were analyzed using Kruskal-Wallis’s test; further analysis was performed by Mann–Whitney (U) test to compare each two groups. *p*-value < 0·05 was considered significant. Correlation coefficient (r) was performed. To assess intra-observer variability, the same observer (O.E) performed the echocardiographic examination at an interval of 1 week to avoid recall bias. To assess inter-observer variability, the echocardiographic examinations were performed by a second observer (D.E) who was blinded to the results of the first observer.

## Results

**Table 1:** Demographic data of the subjects studied

	Cases (*n* = 30)		Control (*n* = 30)		*p*
	No.	%	No.	%	
**Sex**					
Male	23	76.7	24	80.0	0.754
Female	7	23.3	6	20.0	
**Age (years)**					
Min.– Max.	1.0–18.0		1.0–14.0		0.630
Median (IQR)	4.50 (2.50–10.0)		5.50 (3.0–10.0)		
**Consanguinity**					
Negative	4	13.3	27	90.0	< 0.001^*^
Positive	26	86.7	3	10.0	
**Family history of similar condition**					
Negative	13	43.3	30	100.0	< 0.001^*^
Positive	17	56.7	0	0.0	

Table 1 presents key demographic variables (sex, age, consanguinity, and family history). There was no significant difference between patients and control group regarding age and sex. However, there was a significant difference between patients and controls regarding the presence of positive consanguinity and similar conditions in the family highlighting the genetic basis of LSDs.

**Table 2:** Distribution of the patients studied according to their type of LSD (*n* = 30):

	No.	%
MPS	22	73.3
-Type 1 MPS	**4**	**13.3**
-Type 2 MPS	3	10.0
-Type 3 MPS	6	20.0
-Type 4 MPS	9	30.0
**Sphingolipidosis**	**7**	**23.3**
-Gaucher disease	5	16.6
-Nieman Pick disease	1	3.3
-Farber disease	1	3.3
**Free Sialic acid storage disease**	**1**	**3.3**

Table 2 shows that 22 (73.3%) of the studied patients were diagnosed with mucopolysaccharidosis, 7 (23.3%) of the studied patients were diagnosed with sphingolipidosis and 1 (3.3%) was diagnosed with free sialic acid storage disease.

**Table 3:** Summary of enzyme replacement therapy among LSDs patients in this study

Disease	Total Number	ERT
**MPS**		
Type 1 Type 2 Type 3 Type 4	4 3 6 9	4 3 0 9
**Sphingolipidosis**		
Gaucher disease Neiman Pick disease Farber disease	5 1 1	5 0 0
**Free sialic acid storage disease**	1	0

**MPS;** mucopolysaccharidosis

Regarding MPS patients, all patients with MPS type 1, MPS type 2, and MPS type 4 are receiving their specific enzyme replacement therapy. There is no enzyme replacement therapy available yet for MPS type 3 (Sanfilippo), Neiman-Pick disease (till the time of the study), Farber disease, and free sialic acid storage disease **as seen in** Table 3.

**Table 4:** Comparison between the patients studied and controls according to the basal serum MDA and NT-proBNP

Basal laboratory markers	Cases (*n* = 30)	Control (*n* = 30)	t	*p*
**NT- proBNP (pg/ml)**				
Min.– Max.	313.0–506.0	170.0–288.0	16.902^*^	< 0.001^*^
Mean ± SD.	433.63 ± 51.74	231.17 ± 40.34		
**MDA (nmol/ml)**				
Min.– Max.	5.10–6.30	2.50–3.70	34.085^*^	< 0.001^*^
Mean ± SD.	5.67 ± 0.32	2.97 ± 0.29		

NT-proBNP: N-terminal pro-brain natriuretic peptide, MDA: malondialdehyde

SD: **Standard deviation**

t: student t-test

p: *p*-value for comparing the studied groups

*: Statistically significant at *p* ≤ 0.05

Table 4 shows that the mean value of serum N-terminal proBNP and malondialdehyde were significantly higher in patients than in controls, (*P*-value < 0.001).

**Table 5:** Tissue doppler imaging parameters of the studied patients and controls:

	Cases (*n* = 30)	Control (*n* = 30)	Test of Sig.	*p*
**LV S (cm)**				
Min.– Max.	3.0–9.0	6.0–8.0	t = 3.362^*^	0.002^*^
Mean ± SD.	5.77 ± 1.55	6.80 ± 0.66		
**Mitral E’/A’**				
Min.– Max.	0.53–1.30	1.10–1.90	U = 16.0^*^	< 0.001^*^
Median (IQR)	0.78(0.66–0.88)	1.50 (1.40–1.70)		
**MPI**				
Min.– Max.	0.30–0.95	0.30–0.50	U = 297.0^*^	0.017^*^
Median (IQR)	0.40 (0.40 − 0.50)	0.40 (0.30–0.40)		

**(LV S =** mitral annulus peak velocity during ventricular systole, **È=**peak velocity during early ventricular diastole, **À=** Peak velocity during atrial contraction; **MPI** = myocardial performance index)

**Table 6:** Speckle tracking echocardiography parameters of the studied patients and controls

	Cases (*n* = 30)	Control (*n* = 30)	Test of Sig.	*p*
**2D LS**				
Min.– Max.	-25.0– -4.0	-27.0– -20.0	t = 7.067^*^	< 0.001^*^
Mean ± SD.	-16.10 ± 5.27	-23.30 ± 1.84		
**3D-GLS%**				
Min.– Max.	-25.0– -4.0	-25.0– -17.0	t = 4.330^*^	< 0.001^*^
Median (IQR)	-16.10 ± 5.27	-20.63 ± 2.27		
**3D-GCS%**				
Min.– Max.	-25.0– -7.0	-23.0– -15.0	U = 154.0^*^	< 0.001^*^
Median (IQR)	-12.0(-16.0– -8.0)	-18.0(-21.0– -16.0)		
**3D-GAS%**				
Min.– Max.	-33.0–10.0	-29.0– -20.0	t = 4.672^*^	< 0.001^*^
Median (IQR)	-18.80 ± 5.30	-23.97 ± 2.93		
**3D-GRS%**				
Min.– Max.	18.0–50.0	30.0–45.0	U = 170.50^*^	< 0.001^*^
Median (IQR)	25.0 (22.0–35.0)	38.0 (33.0–40.0)		

**2D LS =** two-dimensional longitudinal strain, **GLS**: global longitudinal strain, **GCS**: global circumferential strain, **GRS**: global radial strain, GAS: global area strain

### Echocardiographic data in the included subjects

As regards echocardiographic examination of studied subjects, the mean value of left ventricular systolic mitral annulus velocity (S), and the median of mitral É/Á ratio measured by tissue Doppler imaging were significantly lower in studied patients as compared to healthy controls. Moreover, there was a statistically significant increase in left ventricular myocardial performance index measured by tissue Doppler imaging in the patients’ group compared to the healthy control group **as seen in** Table 5.

Furthermore, there was a statistically significant decrease in the mean value of the 2D longitudinal strain, three-dimensional global longitudinal strain, three-dimensional global radial strain, three-dimensional global circumferential strain, and three-dimensional global area strain in the patients ‘group as compared to the healthy control group. (*p*-value < 0.001) **as seen in** Table 6.

**Table 7:** Distribution of the studied cases according to treatment with CoQ10 (*n* = 30):

Treatment	No.	Dropped cases	No.	%
**Placebo**	**15**			
-MPS -Sphingolipidosis	11 4	**2**	**13**	**43.33**
**Coenzyme Q10**	**15**			
-MPS -Sphingolipidosis -Free sialic acid storage disease	11 3 1	**2**	**13**	**43.33**

Table 7 shows that 15 patients received treatment with CoQ10 *(11 MPS patients*,* 3 patients with sphingolipidosis*,* and 1 with free sialic acid storage disease*) and 15 patients received a placebo *(11 MPS patients and 4 patients with sphingolipidosis).* But unfortunately, four patients dropped off the study, one patient died, and 3 patients didn’t continue the follow-up. So, the final number of patients in each group was 13 patients.

**Table 8:** Comparison between the CoQ10 group and the placebo group of patients regarding their serum NT-proBNP and serum MDA before and after treatment. (*n* = 26):

		Treatment		Test of sig.	*p*
		Placebo group (*n* = 13)	CoQ10 group (*n* = 13)		
***N*** **-terminal proBNP**	**Baseline**				
	Min.– Max.	369.0–498.0	313.0–506.0	t = 0.052	0.959
	Mean ± SD.	431.38 ± 42.92	430.31 ± 60.58		
	**End of follow up**				
	Min.– Max.	395.0–556.0	179.0–480.0	t = 5.113^*^	< 0.001^*^
	Mean ± SD.	451.23 ± 46.0	310.23 ± 88.15		
	**t**_**1**_**(p**_**1**_)	3.870^*^(0.002^*^)	5.476^*^(< 0.001^*^)		
	**Decrease**				
	Min.– Max.	-58.0– -2.0	-11.0–256.0	U = 7.0^*^	< 0.001^*^
	Median (IQR)	-10.0	118.0		
**MDA**	**Baseline**				
	Min.– Max.	5.30–6.30	5.10–6.30	t = 0.503	0.620
	Mean ± SD.	5.67 ± 0.32	5.60 ± 0.34		
	**End of follow up**				
	Min.– Max.	5.50–6.50	3.40–5.0	t = 13.135^*^	< 0.001^*^
	Mean ± SD.	5.94 ± 0.32	4.08 ± 0.39		
	**t**_**1**_**(p**_**1**_)	3.530^*^(0.004^*^)	13.667^*^(< 0.001^*^)		
	**Decrease**				
	Min.– Max.	-1.0– -0.10	0.40–2.0	U = 0.0^*^	< 0.001^*^
	Median	-0.10	1.60		

t: student t-test t_1_: paired t-test U: Mann Whitney test Z: Wilcoxon signed ranks test

p: *p*-value for comparing the studied groups

p_1_: *p*-value for comparing **Pretreatment and Post-treatment**

*: Statistically significant at *p* ≤ 0.05

Table 8 shows a non-significant difference between the placebo group and the CoQ10 group of patients regarding their pretreatment mean values of serum N-terminal proBNP and serum MDA, *P*-value > 0.05. However, there was a significant difference between their posttreatment mean values, *P*-value < 0.001.

There was a statistically highly significant decrease in the posttreatment mean values of MDA and N-terminal proBNP for the CoQ10 group, while there was a statistically significant increase in the posttreatment mean values of MDA and N-terminal BNP for the group that received no cardioprotective treatment, P_1_ value < 0.05.

**Table 9:** Comparison between the placebo and the CoQ10 groups of patients regarding left ventricle tissue doppler echocardiographic parameters before and after treatment (*n* = 26):

	Tissue doppler of LV	Treatment		Test of sig.	*p*
		Placebo group (*n* = 13)	CoQ10 group (*n* = 13)		
**LV S (cm/sec)**	Baseline			t = 0.384	
	Min.– Max.	3.0–8.0	4.0–9.0		0.704
	Mean ± SD.	6.0 ± 1.53	5.77 ± 1.54		
	**End of follow up**				
	Min.– Max. Mean ± SD.	4.0–8.0 6.0 ± 1.35	5.0–8.0 6.31 ± 1.18	t = 0.617	0.543
	**(p**_**1**_)	**(1.000)**	**(0.327)**		
	**Increase**			U = 64.0	
	Min.– Max.	-2.0–2.0	-4.0–3.0		0.311
	Median	0.0	1.0		
**Mitral E’/A’**	**Baseline**	0.56–1.30 0.70	0.53–1.20 0.80	U = 75.50	0.545
	Min.– Max. Median				
	**End of follow up** Min.– Max. Median	0.44–1.10 1.10	0.61–1.60 1.10		0.204
				U = 59.50	
	**Z (p**_**1**_)	**1.119(0.263)**	2.668*(0.008*)		
	**Increases**				
	Min.– Max.	-0.43–0.54	-0.19–0.69	U = 64.50	0.311
	Median.	0.07	0.28		
**LV MPI**	**Baseline**			U = 54.50	
	Min.– Max.	0.30–0.73	0.30–0.95		0.125
	Median	0.40	0.50		
	**End of follow up**				
	Min.– Max.	0.30–0.60	0.30–0.50	U = 35.50^*^	0.010*
	Median	0.50	0.40		
	**Z(p**_**1**_)	**1.389(0.165)**	2.266*(0.023*)		
	**Decrease**				
	Min.– Max.	-0.20–0.43	-0.10–0.65	U = 33.0^*^	0.007*
	Median	-0.10	0.10		

**LV S**: left ventricle systolic mitral annulus velocity, **Mitral E’/A’**: early diastolic mitral annulus velocity/ atrial (late) mitral annulus velocity, **LV MPI**: left ventricular myocardial performance index

t: student t-test t_1_: paired t-test U: Mann Whitney test Z: Wilcoxon signed ranks test

p: *p*-value for comparing the studied groups

p_1_: *p*-value for comparing **Pretreatment and Post-treatment**

*: Statistically significant at *p* ≤ 0.05

**Fig. 1:**
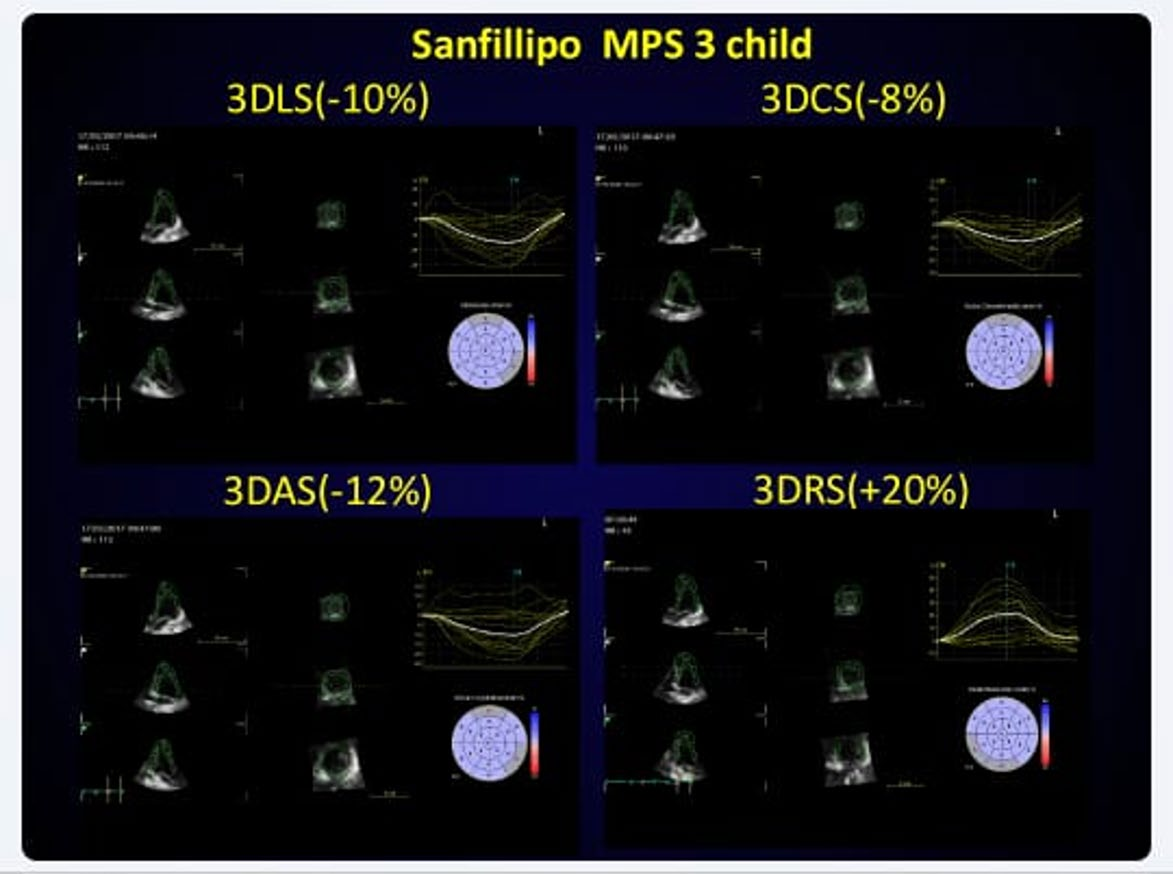
3D Strain Analysis in a Sanfillipo MPS 3 Child Before Treatment. This figure demonstrates the 3D strain analysis in a child with Sanfillipo MPS 3 before enzyme Q10 treatment. The upper left quadrant displays affected 3D longitudinal strain (-10%), the upper right quadrant shows affected 3D circumferential strain (-8%), the bottom left depicts affected 3D area strain (-12%), while the bottom right demonstrates preserved 3D radial strain. These findings suggest significant strain abnormalities, which may have implications for disease progression and response to therapy. (Videos are available)

**Fig. 2:**
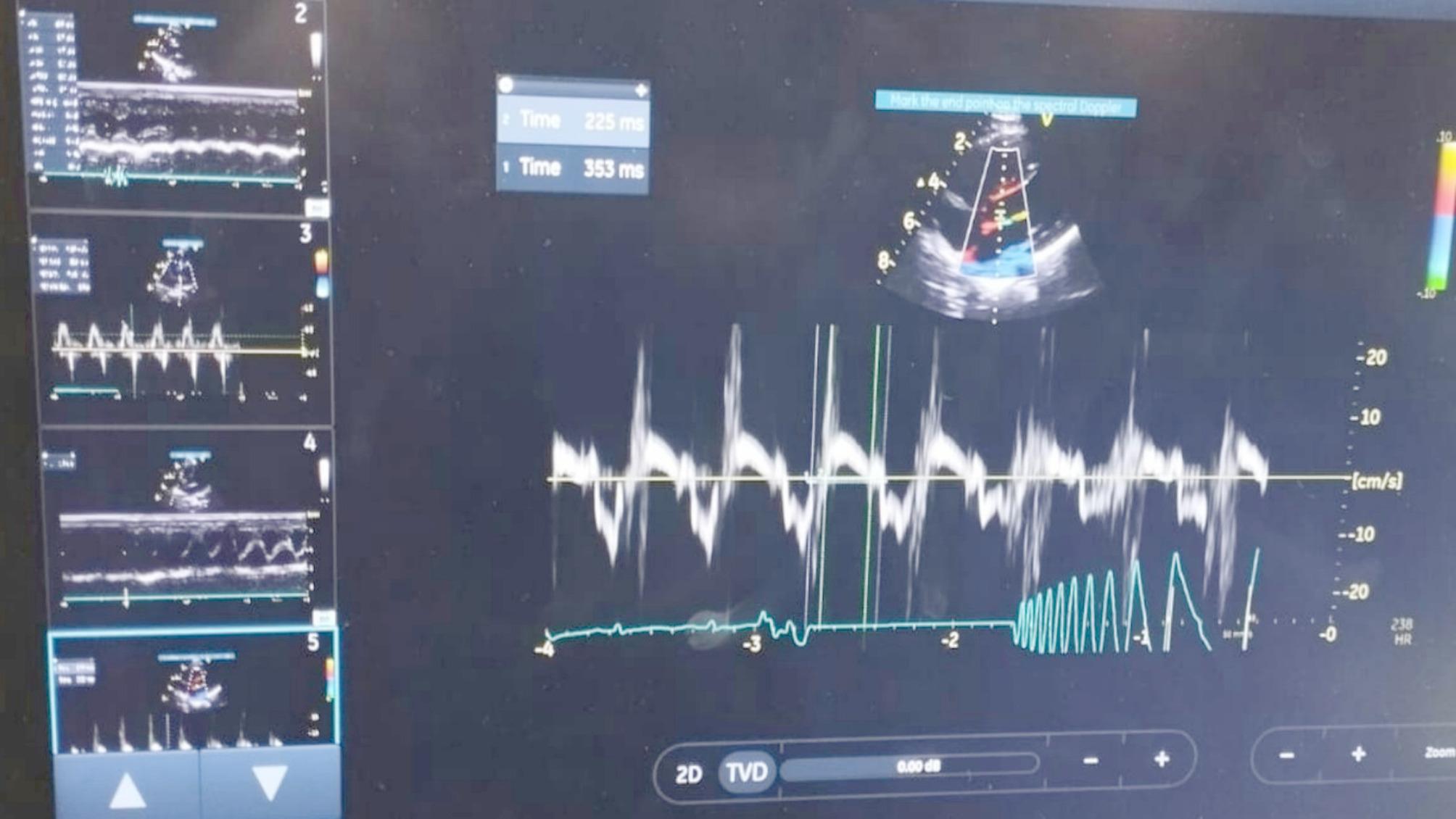
Pulsed Wave Tissue Doppler Imaging Before Treatment. Pulsed wave tissue Doppler imaging from the apical 4-chamber view, sampling from the septal mitral annulus before treatment. The E’/A’ ratio is 0.8, and the left ventricular myocardial performance index (LV MPI) is 0.56 (353 − 225/225). This indicates impaired myocardial relaxation and systolic dysfunction, characteristic of patients with Sanfillipo MPS 3

**Fig. 3:**
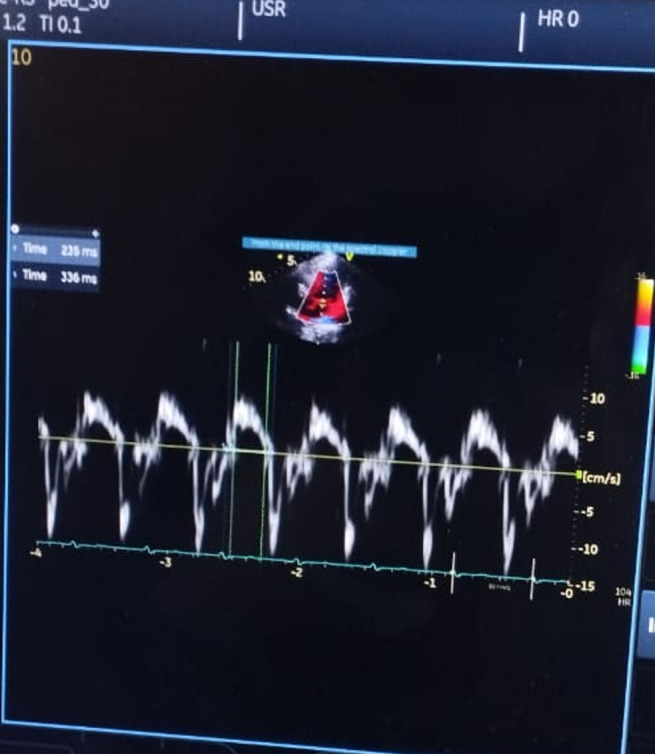
Pulsed Wave Tissue Doppler Imaging After Treatment. Pulsed wave tissue Doppler imaging from the apical 4-chamber view, sampling from the septal mitral annulus in the same child after enzyme Q10 treatment. The E’/A’ ratio has improved to 1.5, and the LV MPI has decreased to 0.42 (336 − 235/235), suggesting an improvement in diastolic and systolic function. This demonstrates the potential beneficial effects of enzyme Q10 therapy in this patient population

Table 9 shows no significant difference between the CoQ10 group and the placebo group of patients regarding their baseline values of left ventricle tissue Doppler echocardiographic parameters (LVS, Mitral E’/A’ and MPI), *p*-value > 0.05. Also, there was no significant difference between both groups regarding their end of follow-up values of left ventricle echocardiographic parameters (LVS, and Mitral E’/A’), *p* value > 0.05. Example of echocardiographic findings before and treatment with enzyme Q10 can be seen in (Figs. 1, 2 and 3).

However, there was a statistically significant decrease of MPI measured by TDI at the end of follow-up in the group that received CoQ10, *P*-value < 0.05 with no significant change occurred in the group who received no cardioprotective drug.

There was no significant difference between baseline and end of follow-up values for each group regarding the LVS parameter, P1 value > 0.05, unlike Mitral E’/A’ which showed a significant increase in the group received CoQ10.

**Table 10:** Comparison between the CoQ10 group and the placebo group of patients regarding left ventricle speckle tracking echocardiography (4D-GLS%) before and after treatment (*n* = 26):

		Treatment		Test of sig.	*p*
		Placebo group (*n* = 13)	CoQ10 group (*n* = 13)		
		Speckle tracking echocardiography			
**4D-GLS%**	**Baseline**				
	Min.– Max.	13.0–25.0	4.0–20.0	t = 1.794	0.087
	Mean ± SD.	18.23 ± 4.28	14.62 ± 5.87		
	**End of follow up**				
	Min.– Max.	10.0–23.0	11.0–25.0	t = 2.240^*^	0.035^*^
	Mean ± SD.	15.92 ± 4.25	19.62 ± 4.15		
	**t**_**1**_**(p**_**1**_)	**1.440(0.150)**	2.266^*^(0.023^*^)		
	**Increase**				
	Min.– Max.	-10.0–3.0	-7.0–14.0	U = 24.0^*^	0.001^*^
	Median	-2.0	5.0		

**4D-GLS**: Four-dimensional global longitudinal strain

t: student t-test t_1_: paired t-test U: Mann Whitney test Z: Wilcoxon signed ranks test

p: *p*-value for comparing the studied groups

p_1_: *p*-value for comparing **Pretreatment and Post-treatment**

*: Statistically significant at *p* ≤ 0.05

Table 10 shows no significant difference between the CoQ10 group and the placebo group of patients regarding their pretreatment speckle-tracking echocardiographic parameters (4D-GLS), *P*-value > 0.05, but there was a significant difference regarding their post-treatment values, *P*-value < 0.05.

There was a significant improvement of 4D-GLS in patients after treatment with CoQ10 with no significant change occurring in the group that received no cardioprotective treatment.

## Discussion

LSDs are characterized by progressive accumulation of undigested material inside lysosomes leading to cellular dysfunction in multiple organs. As a result, a variety of pathogenic cascades are activated such as oxidative stress, inflammation, altered lipid trafficking, and autophagy. It is presumed that metabolites accumulated in LSDs cause an increase in lysosomes’ number and size, which may induce excessive production of reactive species and/or deplete the tissue antioxidant capacity, leading to damage to biomolecules (lipids, proteins, and DNA). It was evidenced that oxidative stress occurs in LSD, even when patients are undergoing enzyme replacement therapy [9].

Moreover, oxidative stress plays an important role in the pathogenesis of cardiovascular diseases. This can be illustrated by the effect of oxidative stress on the phospholamban and SERCA that may lead to impairing diastole or systole (cardiomyopathy and failure).

Phospholamban is a key regulator of cardiac contractility and modulates SR Ca2 + sequestration by inhibiting the SR Ca2+-ATPase (SERCA) in its dephosphorylated state. Upon phosphorylation, which is mediated through beta-adrenergic stimulation, the inhibitory effect of phospholamban on the function of SERCA is relieved. (Frank, K. and Kranias, E.G., 2000. Phospholamban and cardiac contractility [24]. 

The sarco(endo)plasmic reticulum calcium-ATPase (SERCA) is an ion-motive transporter that establishes intracellular calcium (Ca^2+^) stores needed for cell signaling and normal cardiac myocyte function. During the cardiac cycle, sequestration of Ca^2+^ by SERCA2a during diastole is the fundamental mechanism for initiation of cardiac muscle relaxation. Moreover, the rate of SERCA Ca^2+^ uptake is one of the main determinants of the size of the Ca^2+^ store, so SERCA is also critical for regulating the strength of cardiac contraction during systole. Because of its key role in determining cardiac inotropy (contractility) and lusitropy (relaxation), SERCA2a activity is closely governed by an inhibitory interaction with its regulatory partner phospholamban (PLB), a 52-residue single span transmembrane peptide. PLB inhibition of SERCA is relieved by phosphorylation of PLB through protein kinase A (PKA)-dependent adrenergic signaling, increasing SERCA activity to meet increased physiological demand. A complementary mechanism for relief of inhibition is PLB phosphorylation by Ca^2+^/calmodulin-dependent protein kinase II (CaMKII). This pathway is activated by the elevation of cytosolic Ca^2+^ that accompanies increased pacing frequency during exercise or stress [25]. 

Hongyang Xu et al., found that Oxidative stress impacts SERCA function. Commonly, oxidative stress is defined as the imbalance between the generation of pro-oxidants and antioxidant response, leading to an accumulation of oxidized molecules. In their study they have the aging as an example for increasing oxidative stress, and in turn, the SERCA pump has been shown to be susceptible and sensitive to oxidative modifications. The first report that a reduced SERCA activity occurs in biological aging demonstrated selective oxidation on cysteine residues of SERCA proteins. More specifically, cysteine 674 was found to have an irreversible oxidation effect mediated by elevated hydrogen peroxide treatment. In cardiac muscle, oxidation on Cys674 dramatically reduced the SERCA activity and, importantly, impaired the cardiac myocyte relaxation in aged mouse heart. Genetic modification to replace the Cys674 with serine (Cys674Ser) resulted in fewer SERCA inactivation in response to oxidation by ROS [26]. 

Uniformly, all the cardiac issues presenting in the LSDs occur earlier than expected in the general population and worsen progressively [7]. 

Given the role that oxidative stress plays along with its deleterious effects in patients with LSDs and cardiac diseases, antioxidant therapy may be an attractive therapeutic strategy for its additional treatment in the future [9]. 

### CoQ10 in lysosomes

It has been reported to be present in the lysosomal membrane in high concentrations where it is has been proposed to play an important role in maintaining the structure and function of the lysosomes by.

- Participating in the lysosomal respiratory chain; CoQ10 functions as both an electron and proton carrier in lysosomes. Recently it was suggested that lysosomes contain an NADH-dependent CoQ10 reductase involved in the translocation of protons into the lysosomal lumen.
- The acidic environment of the lysosomal lumen (pH; 5.2–6.1) is maintained mainly by a V-ATPase. This enzyme uses the free energy released from the hydrolysis of ATP to pump protons into the lumen of the organelle. So, lysosomal acidification requires functioning mitochondria with adequate CoQ10 to provide the required ATP for this process.
- Has a membrane-stabilizing effect on lysosomal membranes through its antioxidant function.
- Having a role in restoring calcium homeostasis in lysosomes [10] [12, 14, 27, 28].

### Dietary CoQ10 supplements

have received a great deal of attention for the prevention of cardiovascular diseases as it is often deficient in cardiovascular patients. The clinical experience of CoQ10 in cardiology includes studies on heart failure, hypertension, coronary artery disease, diastolic dysfunction of the left ventricle, and ischemia-reperfusion injury [28]. 

Oxidative stress plays a key role in the pathogenesis of CVDs like heart failure and hypertension. In heart failure, there is impaired contractile function due to the mitochondrial energy depletion status which is associated with low endogenous CoQ10 levels (in serum and myocardial tissue samples) in patients with chronic heart failure. Moreover, deficiency of CoQ10 correlated with the severity of the disease, indicating that treatment with CoQ10 may lead to improving the myocardial contractility of cardiac patients [29]. 

Several studies have investigated the benefits of CoQ10 supplements in patients with *cardiovascular risk factors* (for improving their cardiovascular functions via improving energy production & contractility of cardiac muscles, and its potent antioxidant activity). It was demonstrated that CoQ10 has a positive impact on heart performance in congestive heart failure of various etiologies, ischemic heart disease, and a clinically significant blood pressure-lowering effect. These benefits may be explained as Q10 acts by (a) Improving cardiac bioenergetics. (b) scavenging free radicals with antioxidant action. (c) Improving endothelial function with vasodilation. (d) Reducing pro-inflammatory cytokines. (e) Stabilizing membrane action. (f) Preserving myocardial Na+/K + ATPase pump. (g) Aith anti-viscosity effect [28, 30]. 

The underlying cause of *mitochondrial dysfunction* in LSDs appears to be multifactorial, though impaired mitophagy, oxidative stress, and the accumulated aggregates or macromolecules appear to be common inhibitory mechanisms shared between these various disorders. Once impaired, dysfunctional mitochondria may impact the function of the lysosome by the increased generation of ROS, as well as depriving the lysosome of ATP which is required by the V-ATPase proton pump to maintain the acidity of the lumen.

Given the reported evidence of mitochondrial dysfunction in LSDs together with the important symbiosis between these two organelles, therapeutic strategies targeting both lysosome and mitochondrial dysfunction may be a key consideration in the treatment of LSDs [10, 31]. 

Demographic data included patients and controls **summarized in ‘**Table 1**’**. LSD patients included in this study were suffering from MPS I, II, III or IV, Gaucher disease (chronic neuronopathic and non-neuronopathic), Neimann-pick disease type A, Farber disease or free sialic acid storage disease see ‘Table 2’. Summary of enzyme replacement therapy of patients is summarized in ‘Table 3’.

The factors responsible for causing secondary CoQ10 deficiency in LSDs are currently not fully understood but maybe caused by certain conditions like vitamin B6 deficiency (an important cofactor for CoQ10 biogenesis) as described in MPS patients, or may be caused by high levels of oxidative stress as it induces degradation of CoQ10 as suggested by pervious literature, also oxidative stress may inhibit enzymes responsible for CoQ10 biosynthesis [32, 33]. 

CoQ10 clinical benefits are mainly due to its ability to improve ATP production, antioxidant activity (preventing the formation of lipid peroxides and LDL oxidation and this protects against.

atherosclerosis), and membrane-stabilizing properties. These effects are beneficial in not only the treatment but also the prevention of cardiac disorders [10, 17]. 

This study was performed to evaluate oxidative stress in patients with LSDs and to study the possible effects of Coenzyme Q10 as a cardioprotective and an antioxidant drug in those patients.

The measurement of MDA has been broadly used as an oxidative stress parameter of lipid peroxidation [34, 35]. 

In this study, there was a statistically significant increase in serum MDA in the patient group in comparison to the control group denoting increased oxidative stress in patients with LSDs. Also, there was a significant increase of serum Nt-proBNP in patients compared to controls see Table 4’.

This result is in concordance with the study performed by Filippon, et al., on patients with MPS type II for the evaluation of their oxidative stress status. They found that the level of MDA was increased in patients with MPS type II before and during the first 6 months of enzyme replacement therapy when compared to healthy controls [36]. 

Also, Kartha, et al. performed a study to evaluate oxidative stress in patients with Gaucher disease in comparison to healthy controls. They reported a significant increase in serum MDA in participants with Gaucher disease compared to healthy controls [37]. 

Regarding the antioxidant activity of CoQ10, in this study, there was a statistically significant decrease of serum MDA in patients after the intake of CoQ10 for 24 weeks at a dose of 2 mg/kg/day while there was a significant increase in MDA level in the group of patients who received placebo see' Table 8 ’.

These results are like results obtained by Bakhshayeshkaram et al. in their metanalysis study for the evaluation of the effects of coenzyme q10 supplementation on the metabolic profiles of adult patients with chronic kidney disease for a duration ranging from 4 weeks to 17 weeks in a dosage varied from 30 to 200 mg/day. They demonstrated promising effects of CoQ10 supplementation for reducing total cholesterol, LDL-cholesterol, MDA, and creatinine levels in patients diagnosed with chronic kidney disease indicating that CoQ10 treatment changed the oxidant/antioxidant balance in favor of antioxidants [38]. 

Also, Kumar et al. reported similar results as they evaluated the effect of CoQ10 in adult patients with cardiac disease and hypertension. They reported that the myocardium of patients with heart failure demonstrated increased oxidative stress which can be corrected by CoQ10 supplementation. CoQ10 intake resulted in a significant decline in MDA which is an indicator of oxidative stress, indicating that scavenging of free radicals may be a possible mechanism for the beneficial effect of CoQ10 in heart failure [39]. 

In this study, there was a significant decrease in serum NT-proBNP in the group of patients after treatment with CoQ10 for 24 weeks in comparison to the group of patients who received a placebo see ‘Table 8 ‘.

This result agrees with the results obtained by Khan et al. in their double-blinded, randomized controlled trial on 123 patients. Patients were screened during their preoperative vascular clinic appointment and randomly assigned to CoQ10 (400 mg per day) versus placebo for 3 days before surgery. Patients with preoperative cardiac risks included ischemic heart disease, CHF, stroke, and diabetes mellitus. They reported that preoperative administration of oral CoQ10 therapy in a dose of 400 mg/day for 3 days before elective vascular surgical procedures resulted in lowers perioperative NT-proBNP levels and this favors the outcome. (Khan et al., 2020) Similar results were reported by Chen et al [40]

Also, Chen, et al. in their study performed for the evaluation of the effect of liquid CoQ10 supplementation on cardiac function in pediatric dilated cardiomyopathy, reported that it resulted in a slight decrease in the level of NT-proBNP after 24 weeks of supplementation [41]. 

Regarding the role of CoQ10 on the heart, the present study revealed that CoQ10 supplementation in patients with LSDs resulted in no significant difference in echocardiographic TDI parameters (LVS, and Mitral E’/A’), however, there was a statistically significant decrease in MPI measured by TDI and a statistically significant increase of LV 4D-GLS by STE after treatment with CoQ10 compared to the placebo group that showed no significant difference see Table ‘9 and Table 10’.

Mortensen et al. in their study on patients with chronic heart failure reported that CoQ10 supplementation (300 mg/day) is safe, improves symptoms, and reduces major adverse cardiovascular events [42]. 

Kocharian et al. evaluated the effects of CoQ10 in children with idiopathic dilated cardiomyopathy in a randomized, double-blind trial at a dose of 2 mg/kg/day. They evaluated 38 patients with idiopathic dilated cardiomyopathy, 17 being randomized to receive coenzyme Q10 and the remaining 21 to receive placebo. In the sixth month of treatment, children randomized to CoQ10 showed significant improvement in diastolic function evaluated by echocardiography compared with a placebo group. They reported that the mean myocardial performance index significantly decreased for those having 6 months of supplementation with CoQ10, while this change was not significant in those having the placebo [43]. 

On the other hand, Turk, et al. in their study to evaluate the effect of CoQ10 supplementation at a dose of 200 mg/day for 8 weeks on diastolic heart functions in hemodialysis patients, reported that there was no significant difference in myocardial performance index between the patients who received CoQ10 and placebo [44]. 

These contradictory results can be due to the difference in the dose, duration, and the formula used of CoQ10 and its bioavailability, the sample size, the nature of the disease, the presence of adjuvant therapy, and the method of evaluation of diastolic dysfunction as some studies used MPI others used E/’A’, deceleration time and isovolumic relaxation time.

## Conclusions

- Patients with LSDs had significantly higher levels of oxidative stress than controls evidenced by the higher level of serum malondialdehyde.
- Oral intake of antioxidant; Coenzyme Q10 in a dose of 2 mg/kg for a duration of 24 weeks resulted in significant beneficial effects on serum malondialdehyde, NT-proBNP, and cardiac functional parameters (myocardial velocities and myocardial strain) in patients with LSDs.

## Data Availability

The datasets used and/or analyzed during the current study are available from the corresponding author on reasonable request.

## Abbreviations

CoQ10: Coenzyme Q10
ERT: Enzyme replacement therapy
LSDs: Lysosomal storage diseases
MDA: Malondialdehyde
MPI: Myocardial performance index
MPS: Mucopolysaccharidosis
NT-proBNP: N-terminal pro-brain natriuretic peptide
STE: Speckle-tracking echocardiography
TDI: Tissue doppler imaging
